# Effect of an office worksite-based yoga program on heart rate variability: A randomized controlled trial

**DOI:** 10.1186/1471-2458-11-578

**Published:** 2011-07-20

**Authors:** Birinder S Cheema, Paul W Marshall, Dennis Chang, Ben Colagiuri, Bianca Machliss

**Affiliations:** 1 School of Biomedical and Health Sciences, University of Western Sydney, Campbelltown, Australia; 2 Centre for Complementary Medicine Research, University of Western Sydney, Campbelltown, Australia; 3 School of Psychology, University of New South Wales, Sydney, Australia; 4 Yoga Synergy, Sydney, Australia

**Keywords:** exercise, training, insulin resistance, diabetes, obesity, cytokines

## Abstract

**Background:**

Chronic work-related stress is a significant and independent risk factor for cardiovascular and metabolic diseases and associated mortality, particularly when compounded by a sedentary work environment. Heart rate variability (HRV) provides an estimate of parasympathetic and sympathetic autonomic control, and can serve as a marker of physiological stress. Hatha yoga is a physically demanding practice that can help to reduce stress; however, time constraints incurred by work and family life may limit participation. The purpose of the present study is to determine if a 10-week, worksite-based yoga program delivered during lunch hour can improve resting HRV and related physical and psychological parameters in sedentary office workers.

**Methods and design:**

This is a parallel-arm RCT that will compare the outcomes of participants assigned to the experimental treatment group (yoga) to those assigned to a no-treatment control group. Participants randomized to the experimental condition will engage in a 10-week yoga program delivered at their place of work. The yoga sessions will be group-based, prescribed three times per week during lunch hour, and will be led by an experienced yoga instructor. The program will involve teaching beginner students safely and progressively over 10 weeks a yoga sequence that incorporates *asanas *(poses and postures), *vinyasa *(exercises), *pranayama *(breathing control) and meditation. The primary outcome of this study is the high frequency (HF) spectral power component of HRV (measured in absolute units; i.e. ms^2^), a measure of parasympathetic autonomic control. Secondary outcomes include additional frequency and time domains of HRV, and measures of physical functioning and psychological health status. Measures will be collected prior to and following the intervention period, and at 6 months follow-up to determine the effect of intervention withdrawal.

**Discussion:**

This study will determine the effect of worksite-based yoga practice on HRV and physical and psychological health status. The findings may assist in implementing practical interventions, such as yoga, into the workplace to mitigate stress, enhance health status and reduce the risk of cardiovascular and metabolic diseases.

**Trial Registration:**

ACTRN12611000536965

URL: http://www.anzctr.org.au/ACTRN12611000536965.aspx

## Background

Epidemiological studies have consistently demonstrated that chronic work-related stress is a significant and independent risk factor for cardiovascular and metabolic diseases and associated mortality [1-4]. For example, a study enrolling over ten thousand participants concluded that individuals with high work-related stress were more than twice as likely to develop metabolic syndrome versus those reporting lower levels of stress [1]. Studies have also noted a strong association between work-related stress and coronary artery disease (CAD) [3,4] and a recent meta-analysis has concluded that work stress can increase the risk of myocardial infarction by 50% [2].

The link between stress and chronic disease is mediated by endocrine pathways of the sympathetic nervous system (SNS) including the hypothalamus-pituatary-adrenal (HPA) axis [5,6]. Cortisol, the main effector of HPA activation, increases circulating fatty acid and glucose concentrations and inhibits insulin. This hormone-mediated pathway prepares the body for physical exertion (i.e. "fight or flight"). The actions of cortisol are particularly concerning in a sedentary work environment given that psychological stress compounded by inactivity induces hyperlipidemia and hyperglycemia, antecedents to more advanced cardiometabolic disease (e.g. hypertension, obesity, type 2 diabetes, CAD, etc.) [7-9]. Interventions that holistically mitigate work-related stress and inactivity may reduce the risk of chronic diseases.

Yoga is an ancient system of practices based on the scientific principles of exercise, breathing and meditation, and philosophical beliefs concerning life and thinking [10]. The origin of yoga has been ascribed to the Indus Valley Civilization (2600-1900 BCE) although some researchers suggest more ancient origins [11,12]. Participation in yoga has increased dramatically throughout the world in recent decades [13]. Many styles of yoga have been tested in the clinical setting and most involve the performance of physical postures (*asanas*), breathing exercises (*pranayama*) and meditation [10]. Randomized controlled trials published in leading medical journals have revealed that yoga can induce significant and broad-ranging health adaptations, both in apparently healthy and chronically diseased cohorts [14-18].

Heart rate variability (HRV) is the beat-to-beat variation in heart rhythm due to autonomic influence on the sinoatrial node. Higher HRV indicates greater parasympathetic control while lower HRV indicates lesser parasympathetic control. Low HRV is an established predictor of cardiovascular morbidity and mortality [19]. Notably, studies have shown that excess job strain reduces HRV [20]. By contrast, two recent trials analysing HRV have demonstrated that participation in yoga sessions can acutely increase HRV [21,22]; however, there are currently no data to suggest that these effects can be maintained long term. An increase in resting HRV secondary to prolonged yoga training would indicate lower physiological stress and reduced risk of chronic diseases and early mortality.

It is oftentimes difficult for individuals to commit to stress-reducing practices due to the time restrictions incurred by work and family life. We postulate that integrating yoga into the workplace could provide a time-effective, convenient and practical method of abating the damaging effects of stress and inactivity. Yoga requires minimal space and minimal investment in equipment, and the potential benefits to health status, wellbeing, job satisfaction and work-related productivity could be significant.

Only two trials have evaluated the effect of a worksite-based yoga program on health-related outcome measures [23,24]. However, neither study evaluated changes in HRV. Therefore, the primary purpose of the present study is to determine the effect of a worksite-based yoga program on resting HRV and associated parameters in a cohort of office workers. We hypothesize that a worksite based yoga program will increase HRV, reflective of greater parasympathetic (vagal) tone, and that this adaptation will be accompanied by improvements in physical fitness and psychological health status, including quality of life, anxiety, and job satisfaction.

## Methods and design

### Study Design

This is a parallel-arm randomized controlled trial that will compare the outcomes of participants assigned to the experimental treatment group (yoga) with those assigned to a no-treatment control group. The primary outcome of this study is the high frequency (HF) spectral power component of HRV (measured in absolute units; i.e. ms^2^), a measure of parasympathetic autonomic control. Secondary outcomes include additional frequency and time domains of HRV, and measures of physical functioning and psychological health status. The intervention period will be 10 weeks, and the assessment of primary and secondary outcome measures will be completed prior to and following the intervention period and at 6 months follow-up to determine the effect of intervention withdrawal. The University of Western Sydney Human Research Ethics committee has approved all research procedures.

### Sample Size and Power Calculation

To our knowledge there are no published data on the effects of yoga training on resting HRV in a non-clinical population. However, it has been shown that yoga induces adaptations that are similar to those achieved with conventional exercise [25]. Therefore, data derived from an aerobic exercise intervention trial in an apparently healthy adult cohort [26] has been used to compute statistical power *a priori *using the non-commercial statistical power analysis program G*Power. The control group is expected to experience no change in the HF spectral power component from baseline (205.7 ± 205.7 ms^2^) while an experimental group is expected to increase this measure following the 10-week yoga intervention (568.0 ± 696.0 ms^2^). Setting an alpha level of 0.05, approximately 56 participants (28 per group) will provide 80% power to detect a statistically significant difference between groups. Recruitment will be inflated to 67 participants to enable a 20% participant attrition rate.

### Participants

Eligibility criteria include: Adult (> 18 years) employed in a full-time academic or general staff office position at the University of Western Sydney; not currently engaged in regular yoga practice; available to attend three yoga sessions per week during lunch break, ability to communicate in English; no acute or chronic medical conditions which would make yoga potentially hazardous or primary outcomes impossible to assess as outlined by the *American College of Sports Medicine *(ACSM) [27]; willingness and cognitive ability to provide written informed consent. Participants will be pre-screened for participation using the *Physical Activity Readiness Questionnaire *[28], and a standardised health history questionnaire. Individuals deemed moderate-risk [27] will require the approval of their physician prior to participation. High-risk individuals [27] will be excluded.

### Randomization

Participants will be randomized *via *computer-generated randomly permuted blocks [29] stratified by gender and age (< 50 years; > 50 years) into an experimental group and no-treatment control group. An investigator not involved in testing or delivery of the intervention will prepare the randomization assignments. Group assignment will be delivered to participants in sealed envelopes upon the completion of baseline testing.

### Interventions

#### Experimental Group

Participants randomized to the experimental condition will engage in a 10-week yoga program delivered at their place of work (University of Western Sydney, Campbelltown Campus). The yoga sessions will be group-based, prescribed three times per week during lunch hour (approximately 50 minutes per session), and will be led by an experienced yoga instructor from Yoga Synergy Pty Ltd (Sydney, Australia) [10]. The program will involve teaching beginner students safely and progressively over 10 weeks a yoga sequence that incorporates *asanas *(poses and postures), *vinyasa *(exercises), *pranayama *(breathing control) and meditation. The sequence design will be aimed at developing strength, flexibility, cardiovascular fitness, and the ability to deal with stress. Many postures incorporate a simple and a more challenging version so participants will be able to choose the level of difficulty that is appropriate to them on any given day. All participants will be advised to change into appropriate clothing prior to arriving at the venue.

#### Control Group

Participants randomized to the control condition will be given education about the benefits of physical activity and relaxation training but will receive no specific instructions about yoga practice for the 10-week intervention period.

### Outcome Measures

All outcome measures will be collected at baseline (week 0) and after the intervention period (week 11), as well as at 6 months follow-up. Qualified and experienced personnel, blinded to the group assignment of participants, will assess the primary and secondary outcomes within one testing session conducted at all three assessment timepoints.

#### Heart Rate Variability

The evaluation of HRV will be completed in a quiet and temperature controlled room in accordance with procedures developed by the *Task Force for Pacing and Electrophysiology *[30]. Participants will be advised to abstain from caffeinated food and beverages on the day of their assessments. Repeat assessments will be completed at precisely the same time of day. After 15 minutes of supine rest with a regular and calm breathing pattern, a continuous 10-minute ECG recording will be collected using an applanation tonometer interface with HRV software (Sphygmocor, AtCor Medical Pty, Sydney, Australia). The primary outcome of this study is the high frequency (HF) spectral power component of HRV (measured in absolute units; i.e. ms^2^). From the electrocardiographic recording, the following statistical and geometric time domain indices will be calculated from RR intervals: standard deviation of the NN intervals (SDNN), and the triangular index (TI). Frequency domain variables, including total, HF and low frequency (LF) power and LF:HF ratio will be derived from spectral analysis of successive R-R intervals. This technique separates the heart rate spectrum into its frequency components and provides quantitative estimates of sympathetic and vagal (parasympathetic) neural influences on the heart.

#### Physical Functioning

Upper-body and abdominal muscular endurance will be evaluated using the standardised push-up test and partial curl-up test, respectively, according to procedures outlined by the *American College of Sports Medicine *[27]. A modified version of the push-up test, allowing the participant to place their knees on the mat, will be used for participants who are unable to complete a single standard push-up [27]. Low back and hip flexibility will be evaluated *via *standardized sit and reach test using equipment and protocols outlined by the *American College of Sports Medicine *[27]. Low back and abdominal muscular endurance will be evaluated by means of an isometric, side-bridge test of muscular endurance [31]. This test demonstrates excellent test-retest reliability in adults (r = 0.99) [31]. Poor trunk muscle endurance has been implicated in the development of chronic low back pain [31].

#### Psychological Health Status

The *Medical Outcomes Trust Short-form 36 Health Status Questionnaire (SF36) *Version 1.0 [32] is a generic health status measure that assesses eight domains of quality of life. This instrument demonstrates a high degree of internal consistency and construct validity, has been designed for self-administration [32], and has been shown to be sensitive enough to detect changes over time in response to a yoga intervention [18,33]. The *State-Trait Anxiety Inventory *is a widely used and validated inventory that consists of two, twenty-item self-report scales for measuring state anxiety and trait anxiety as distinct and clearly defined psychological constructs in adults [34]. Job satisfaction and general work-related stress will be evaluated *via *global *Job Descriptive Index *and *Job in General Scale*, respectively [35]. Both scales have been validated for use in the general population, including office workers [35].

### Demographic and Health Status

Demographic and health status data will be extracted during the recruitment and screening process and baseline testing by means of standard questionnaires and assessments. Factors include height, body weight, resting systolic and diastolic blood pressure, waist circumference, demographic characteristics (i.e. age, gender, occupation, marital status, living arrangement, income, smoking history, alcohol intake, etc.), medication and supplement usage and dosage, adverse events related to medication/supplement intake, and any medical and surgical procedures received. Change of health status and physical activity in the experimental and control group during the 10-week intervention period will be documented by means of a structured questionnaire of open-ended questions that will be administered weekly *via *email. Acute illnesses, falls, changes in medication dosage and usage, adverse events related to yoga participation and visits to health care professionals will be documented in both the experimental and control group. Any participant experiencing an adverse event due to the yoga program will be referred to a qualified health care practitioner for appropriate management. All adverse events will be documented and reported.

### Statistical Analyses

Primary analysis will be via intention-to-treat with all patients included regardless of dropout or level of adherence. Missing data will be imputed according to the maximum likelihood expectation algorithm *via *the Statistical Package for the Social Sciences (IBM©, SPSS Version 19.0). Data will be presented as the mean ± standard deviation or median and range, as appropriate. Confidence intervals will be used to express group differences. Changes between groups will be determined by analysis of covariance of the post-treatment score controlling for the baseline score. Effect sizes and 95% confidence intervals will be calculated. A p value of < 0.05 will be considered indicative of statistical significance; clinical significance will be interpreted in light of the meaningfulness and magnitude of the adaptations observed.

## Discussion

The results of this study will help determine the efficacy of worksite-based yoga practice on HRV and parameters of physical and psychological health status. These findings may assist in implementing practical interventions such as yoga into the workplace to mitigate stress, enhance health status and reduce the risk of cardiovascular and metabolic diseases.

## Competing interests

BSC, PWM, DC and BC declare that they have no competing interests. BM is owner of a commercial Yoga studio, Yoga Synergy Pty Ltd.

## Authors' contributions

BSC designed the study and drafted the manuscript. PWM provided consultation regarding physical functioning outcome measures. DC provided consultation regarding heart rate variability method. BC provided consultation regarding statistics and psychological outcome measures. BM designed the yoga intervention. All authors have read and approved the final manuscript.

## Pre-publication history

The pre-publication history for this paper can be accessed here:

http://www.biomedcentral.com/1471-2458/11/578/prepub
